# Evaluation of *NID2* promoter methylation for screening of Oral squamous cell carcinoma

**DOI:** 10.1186/s12885-020-6692-z

**Published:** 2020-03-14

**Authors:** Ratakorn Srisuttee, Areeya Arayataweegool, Patnarin Mahattanasakul, Napadon Tangjaturonrasme, Virachai Kerekhanjanarong, Somboon Keelawat, Apiwat Mutirangura, Nakarin Kitkumthorn

**Affiliations:** 1 Faculty of Medicine, King Mongkut’s Institute of Technology Ladkrabang, Bangkok, 10520 Thailand; 2 Center of Excellence in Molecular Genetics of Cancer and Human Diseases, Department of Anatomy, Faculty of Medicine, Chulalongkorn University, Bangkok, 10330 Thailand; 3 Department of Otolaryngology, Faculty of Medicine, Chulalongkorn University, Bangkok, 10330 Thailand; 4 Department of Otolaryngology, Head and Neck Surgery, King Chulalongkorn Memorial Hospital, Thai Red Cross Society, Bangkok, 10330 Thailand; 5 Department of Pathology, Faculty of Medicine, Chulalongkorn University, Bangkok, 10330 Thailand; 6 Department of Oral Biology, Faculty of Dentistry, Mahidol University, Payathai Rd., Ratchathewi, Bangkok, 10400 Thailand

**Keywords:** *Nidogen2*, Methylation, Detection, Oral squamous cell carcinoma

## Abstract

**Background:**

Oral squamous cell carcinoma (OSCC) is an aggressive human malignancy. Because of late diagnosis and recurrence of OSCC, the treatment of patients with OSCC is often ineffective. Thus, finding novel biomarkers of OSCC are essential. Here we derived a methylation marker by utilizing methylation microarray data and testing its capacity in cross-sectional study designed for OSCC detection and screening.

**Methods:**

According to bioinformatics analysis of total of 27,578 cg sites, cg22881914 of Nidogen 2 (*NID2*) methylation was selected for evaluation. Next, we confirmed the methylation status by bisulfite sequencing from the microdissected OSCC cells in comparison with the microdissected oral epithelia. Subsequently, we developed a simple technique using real-time PCR with the specific probe to examine the ability for the detection of OSCC in the oral epithelial samples, which included 103 oral rinse and 82 oral swab samples.

**Results:**

Based on the comparison of microdissected tissue, cg22881914 of *NID2* was proved to be methylated in most OSCC cells but unmethylated in the normal oral epithelia. Furthermore, the methylated *NID2*-relied quantitative PCR approach has demonstrated that this marker assists in distinguishing among patients with OSCC from normal oral epithelia, smokers, and patients with oral lichen planus using the non-invasive oral rinse and swab samples.

**Conclusions:**

Specific methylation at cg22881914 of *NID2 *of OSCC could be used as an important potential marker for detecting OSCC. Thus, to certify the utility of this marker, further studies with a larger sample size are needed.

## Background

Oral cancer is a major health issue, with an incidence rate more than 280,000 patients, of which almost 50% died. Notably, oral cancer is more prevalent in men [1–3]. Moreover, the highest incidence rates of oral cancer for both men and women in general are noted in Southeast Asia and Central and Eastern Europe [4]. Histopathologically, most oral cancer cases are clinically classified as squamous cell carcinoma, which is the cancer tissue type found in the nearby organs, such as head and neck and oropharyngeal cancers [5, 6]. In addition, because of the rapid growth of oral squamous cell carcinoma (OSCC), the tumor staging is promptly developed, leading to increased size and distant metastasis. Consequently, this condition is often followed by a decrease in the overall 5-year survival rate to 60% [3].

The treatment options for oral cancer depend on the cancer stage at which it is diagnosed. The main approach is surgery, which is usually considered in use for those who have locally advanced and resectable lesions. On the other hand, in patients with unresectable lesions, radiotherapy and chemotherapy would be a recommended treatment option for oral cancer, particularly metastatic OSCC [7]. To date, several targeted therapies approved by the US Food and Drug Administration have been used to prevent cancer recurrence in the head and neck area, including cetuximab, bevacizumab, PD-1 and mTOR inhibitors, nivolumab, and pembrolizumab [8]. However, the possibility of using of target therapeutic approaches as essential treatment is particularly difficult because of the low rate of successful investigations and high rate of mortality in recurrence cases [9]. Thus, early-stage detection remains essential for treatment.

Tissue biopsy is the gold standard method for the early detection of oral cancer, but such procedures are expensive and invasive, which may cause patient discomfort [10]. As such, potential noninvasive tools have been developed, e.g., saliva test. In particular, more than 120 saliva-based biomarkers have been investigated and improved from the genetic to proteomic levels [11]. However, neither the sensitivity nor specificity levels of these markers have been sufficiently elucidated [5, 12, 13]. In our previous study using a methylation-specific database for OSCC, we revealed two methylated cg sites that can distinguish the differentiated OSCC from normal oral epithelia. One of these was cg01009664 of the *TRH* gene, which was a novel marker for screening. In this study, more interestingly and effectively, we have proposed another screening marker, nidogen 2 (*NID2*), and developed a method better than using cg01009664 of *TRH* for OSCC screening [14].

## Methods

### Ethical statements

The study protocol was approved by the Institutional Review Board (IRB 426/58 and 135/59), and informed consent forms were obtained in patients in the oral rinse and swab groups.

### Bioinformatics

**Fig. 1:**
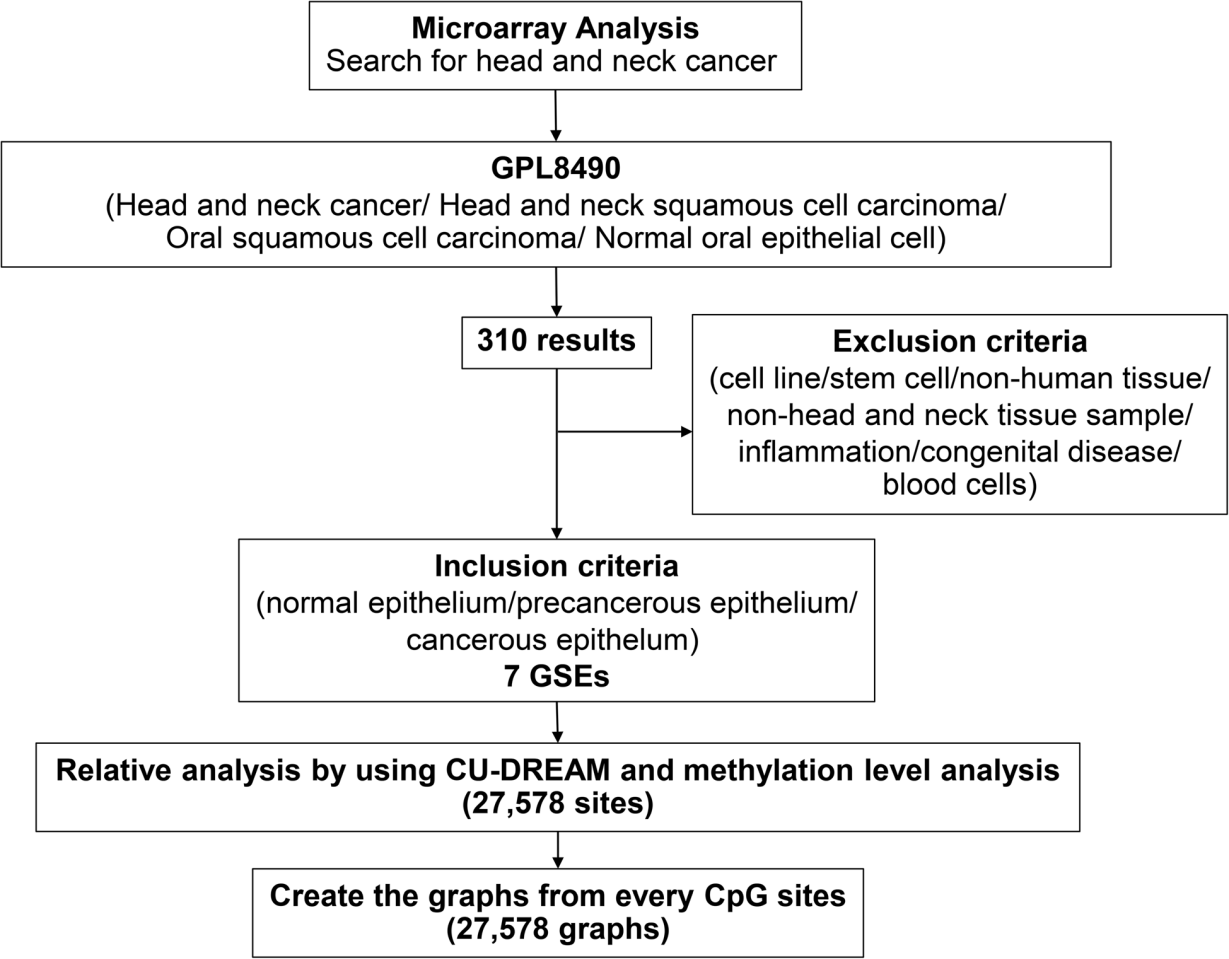
Flowchart of the bioinformatic analysis

The bioinformatic approach was previously reported [14]. In brief, we collected the methylation microarray data of GPL8490 (Illumina® HumanMethylation27 BeadChip Kit, Illumina Inc., San Diego, CA, USA) related to the following keywords: head and neck cancer, head and neck squamous cell carcinoma (HNSCC), OSCC, and normal oral epithelial cell. The inclusion criteria were normal, precancerous, and cancerous epithelia of the head and neck. On the other hand, the exclusion criteria included cell line, stem and blood cell, nonhuman tissue, non-head and neck tissue sample, inflammation, and congenital disease. Ultimately, seven series of experiments (GSEs) were selected. The Connection Up- and Down-Regulation Expression Analysis of Microarrays program was used to calculate the mean value of the methylation percentages of 27,578 cg sites in each sample and then create 27,578 graphs. Each graph displayed the mean value of the methylation percentages of the normal, precancerous, and cancerous cells. Furthermore, *NID2* at cg22881914 was selected as they distinctively distinguished the difference between the normal oral epithelial cell and cancerous cell methylation percentage values. The bioinformatics process is illustrated in Fig. 1.

### Sample recruitment

A total of 20 formalin-fixed paraffin-embedded (FFPE) tissue samples (10 OSCC and 10 normal oral epithelia) were collected from the Department of Pathology, Faculty of Medicine, Chulalongkorn University. The FFPE blocks were recut and stained with hematoxylin and eosin (H&E) for the histopathological review by a certified pathologist (SK). Next, manual microdissection technique was performed according to previously described report [15]. Briefly, 5-μm-thick sections of both OSCC and normal mucosa FFPE blocks were serially cut into five levels. The first and last of the total 5 slides were then stained with H&E. Then, the selected areas (tumor cells in OSCC and normal squamous epithelial cells in the normal mucosa) on the first slides were outlined by a marker pen, and those on the last slides were also marked using the first slides as references. Subsequently, these were examined under the microscope. Then, if the last slides were correctly marked similar to the first slide, the remaining unstained slides (levels 2–4) would be processed in the same manner using the first and last H&E slides as references for area selection. Finally, the selected areas were dissected using sterile needle-gauge 21, microdissected specimens were kept in phosphate-buffered saline (PBS) solution until DNA extraction.

In the oral rinse, the samples from 43 patients with OSCC, 40 smokers, and 50 healthy controls were included. Additionally, in the oral swab collection, there were 22 matched OSCC patients who allowed collecting oral swab, 30 patients with oral lichen planus (OLP), and 50 healthy controls. All volunteers were enrolled in the study were collected from the Department of Otolaryngology, Faculty of Medicine, Chulalongkorn University during January 2016 to December 2017. Sample size of OSCC group was a total of OSCC patients in that period. Detailed data are shown in Table 1. All participants were given a self-administered questionnaire to collect their medical history and information on smoking. After completing the questionnaire, the patients underwent clinical examination by surgeons (PM, NT, VK). The healthy controls were those who had no oral mucosal lesion or history of malignancy and did not smoke after the oral examination and history taking. The healthy controls were randomly selected with matched-age group to OSCC group. Smoking consumption data included number of years smoked and number of cigarettes smoked daily. In addition, the diagnoses of patients with OLP and OSCC were confirmed by the histological findings from incisional biopsy. OLP diagnosis was confirmed by an oral pathologist (SK) using the histological diagnostic criteria of lichen planus set by Kruppa et al. 2015 [16]. OLP was excluded the diagnosis of oral lichenoid reactions based on the medication history, direct contact with dental restorative materials, and history of grafting or graft- versus host diseases [17].

**Table 1:** Demographic data and NID2 methylation levels of the samples

	Healthy control (*n* = 50)	Potentially malignant group (*n* = 70)		Cancer group (*n* = 43)	*P*-value
Age mean (SD)	55.6 (15.3)	58.2 (13.9)	53.8 (15.6)	57.3 (17.5)	0.537
Gender	Male, 54 (54%)	Male, 26 (72.5%)	Male, 13 (36.7%)	Male, 23 (53.5%)	0.345
	Female, 46 (46%)	Female, 14 (27.5%)	Female, 17 (63.3%)	Female, 20 (46.5%)	
Sample collection					
Oral rinse	Healthy controls, 50^a^	Smokers, 40		Oral squamous cell carcinoma, 43	
Oral swab	Healthy controls, 50^a^		Oral lichen planus, 30	Oral squamous cell carcinoma, 22	
Histological grade				Well-differentiated, 24	
				Moderately differentiated, 13	
				Poorly differentiated, 5	
Clinical stage				Stage I, 7	
				Stage II, 10	
				Stage III, 4	
				Stage IV, 22	
Methylated NID2 concentration					
(ng/ul) [mean(SD)]					
Oral rinse	0 (0)	0 (0)		4.33 (7.69)	< 0.001
Oral swab	0 (0)		0 (0)	4.00 (8.34)	< 0.001

^a^ Healthy controls in the oral rinse and oral swab were the same people

The oral epithelial cells were collected using an oral rinse (from patients with OSCC, smokers, and healthy controls) and oral swab (from patients with OSCC, OLP and healthy controls). In the oral rinse, 0.9% normal saline solution was gargled for 15 s, whereas in the oral swab, a foam-tipped applicator (Puritan Medical Products, Maine, USA) was applied over the OSCC, OLP lesion, and normal buccal mucosa of healthy controls for 5–10 s. Then, the oral rinse solutions and oral swab foams were kept in a sterile tube and stored at 4 °C until the DNA extraction process.

### DNA isolation and sodium bisulfite modification

Genomic DNA was extracted from the cell pellet of the oral rinse and swab samples and 10-μm-thick unstained slides (10 slides per sample). The unstained slide samples were prior deparaffinized by xylene. Thereafter, all sample groups were lysed using a lysis buffer (0.75 mol/L NaCl, 0.024 mol/L EDTA, pH, 8.0) that was mixed with 10% sodium dodecyl sulfate and 20 mg/mL proteinase K for digestion, followed by standard phenol–chloroform extraction [15]. Furthermore, the DNA concentration was measured using a NanoDrop and subsequently adjusted to 750 ng/μL. The bisulfite treatment to 20 μL of each sample was undertaken using the EZ DNA Methylation Kit (Zymo Research, CA, USA) according to the protocol guidelines. Then, the converted DNA was eluted in 20 μL of M-Elution Buffer and stored below − 20 °C for subsequent use.

### *NID2* bisulfite sequencing

All microdissected samples were checked for the methylation sequence at cg22881914 of *NID2* by direct sequencing of the PCR products. The forward primer was 5′-GYGYGTAGGTTAGTAGTYGTATT-3′, and the reverse primer was 5′-CCCRAATCATCCTCTCATCCRA-3′.

### *NID2* methylation real-time PCR

To detect methylation at cg22881914 of *NID2*, two real-time PCRs were conducted from bisulfite-modification DNA 35 ng in each PCR reaction. The *NID2* methylation set was composed of the forward and reverse primers 5′-CGTATTCGTCGTTGCGGG-3′ and 5′-CCGAATCATCCTCTCATCCG-3′, respectively, and the probe 5′-Fam-CGTTGAGTTTATTTTTTGTAACGTC-MGB-3′, with an annealing temperature of 59 °C. The *beta-actin* set served as the internal control, for which the forward and reverse primers were 5′-GTGTATTTGATTTTTGAGGAGA-3′ and 5′-CCTTAATACCAACCTACCCAA-3′, respectively, and the probe was Cy5–5’AAGGTGAAYGTGGATGAAGTTGGTGGTGAGG3’BHQ, with an annealing temperature of 59 °C [18]. The real-time PCRs were executed in duplicates with 7500 Fast Real-Time PCR System (Applied Biosystems, Carlsbad, CA, USA).

### *NID2* methylation calculation

The standard curve of methylation set was performed to detect the minimal DNA concentration that could be amplified by the 10-fold dilution of bisulfite DNA from 10 to 1 pg. A serial dilution of the completely methylated DNA (EpiTect® PCR control kit, Qiagen, Hilden, Germany) was prepared in the concentrations of 10 to 1 pg as the standard, which were diluted with unmethylated DNA up to 10 ng/μL in total concentration for the investigation of its sensitivity. The threshold cycle (Ct) value of the methylated *NID2* level in each individual sample was calculated from the standard curve using the following equation: y = 7E + 10e − 0.695x (Fig. 3b). The Ct value of the beta-actin measurement was used as the internal control.

### Statistical analyses

The SPSS software for Windows version 22 (SPSS Inc., Chicago, IL) was used to analyze all data. Moreover, ANOVA was performed to determine the difference between healthy controls, smokers, and patients with OSCC in the oral rinse samples and between healthy controls and patients with OLP and OSCC in the oral swab samples. Also, the effect of age, gender, histological grade, and disease stage on the methylation status was investigated using the Pearson chi-square (χ2) analysis. *P* values < 0.05 were considered statistically significant (two-sided).

## Results

### Discovery of cg22881914 of *NID2* specific to OSCC

**Fig. 2:**
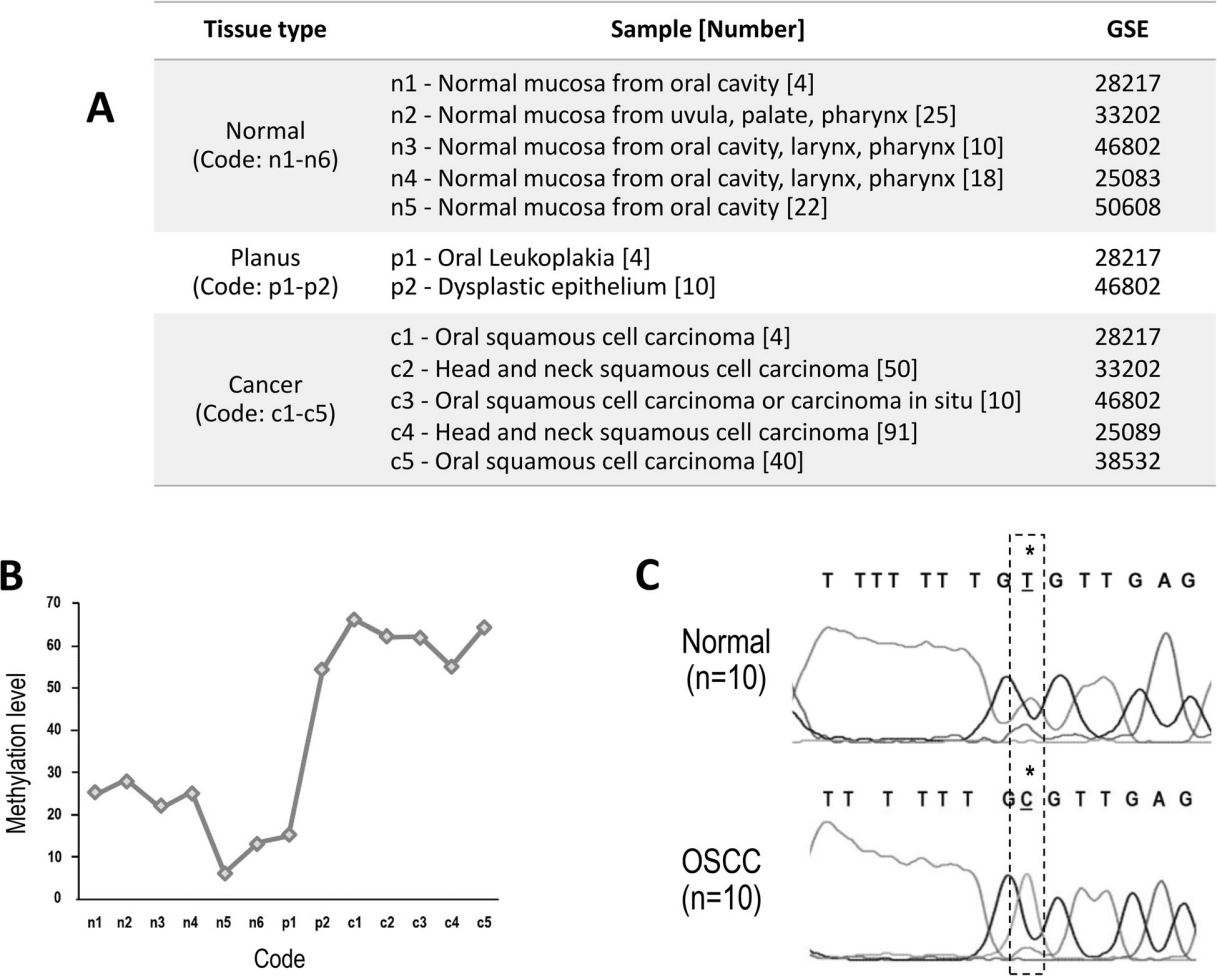
*NID2 *methylation occurred on the cg-specific site in the cancer tissues. **a** Several types of oral tissue samples, including both normal and cancer tissues were collected from the NCBI resources. **b** The methylation levels ranking from normal to carcinoma were evaluated. **c** The bisulfite-converted DNA of the normal and cancer tissues were sequenced on the methylated site of the *NID2* gene position

The bioinformatics data of 27,578 cg comparation sites, cg22881914 of *NID2* displayed prominent differences in the methylation value among healthy controls, premalignant patients, and patients with OSCC (Fig. 2b). To ensure the methylation at cg22881914 in *NID2* presented in OSCC cell, not in normal oral epithelial cell, manual microdissection was performed. Also, bisulfite sequencing was used to evaluate more than 90% of cancer cells in OSCC and more than 90% of the normal oral epithelial cells. Verification with sequencings at cg22881914 of *NID2* exemplified in Fig. 2c, conversion of cytosine to thymine was completed on unmethylated bases but not at methylated bases, suggesting the methylation status in *NID2* of oral cancer.

### Screening of cg22881914 of *NID2* in the oral rinse and swab samples

**Fig. 3:**
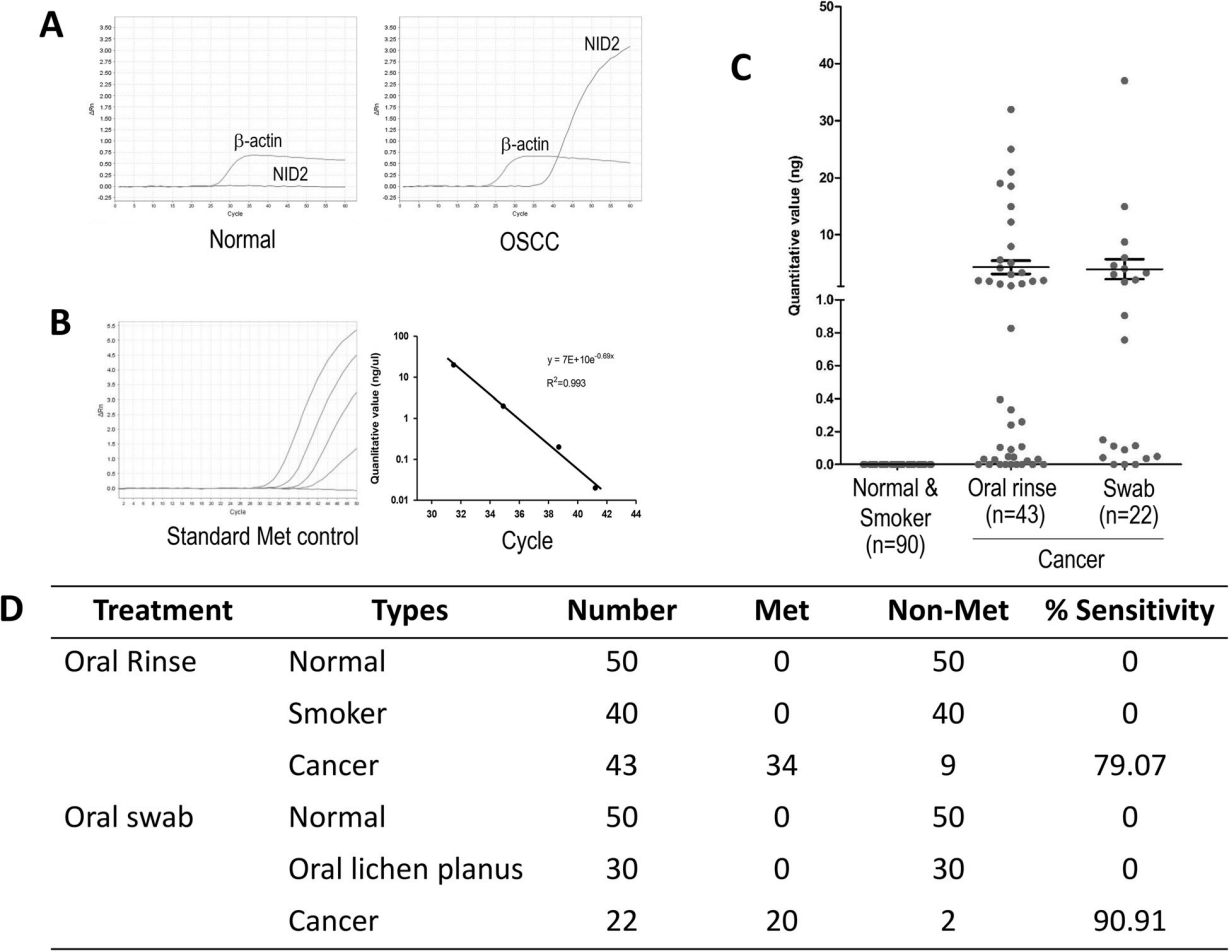
*NID2* methylation level on the specific site is higher in the cancer tissues compared with that in the normal oral epithelium. **a** The bisulfite-converted DNA of the normal oral epithelium and cancer samples were amplified using the quantitative PCR at methylated *NID2* with a specific probe. **b** Universal standard methylation controls were amplified using the real-time PCR (left) and then calculated into standard curve (right). **c** These bisulfite-converted DNA samples from the healthy controls, smokers, and patients with OSCC were amplified with a specific probe, and the methylation level was estimated referring to the standard curve. **d** Comparison of the effective collection approaches between oral rinse and oral swab in detecting *NID2* methylation

To prove the methylation of *NID2*, the samples were collected using oral rinse or oral swab from patients with oral cancer in different grade and stage to compare with healthy control group. Additionally, the samples of those who are at a risk of oral cancer were also included for investigation (Table 1). For screening purposes, we improved our technique by using duplex real-time PCR with Taq man probe, which was able to measure *NID2* and *beta-actin* in a loading (Fig. 3a). The detectability of real-time PCR was performed to minimal DNA concentration at 0.1 ng/μL (Fig. 3b). *NID2* methylation was then tested in the clinical samples, of which the characteristics are given in Table 1.

As shown in Fig. 3c, *NID2* was completely unmethylated (quantitative level = 0 ng) in all samples of the healthy controls, smokers, and patients with OLP for which individual epithelia oral rinse or swab samples were collected. Consequently, we analyzed the methylation data in qualitative instead. With the rule of the methylation quantitative level, more than 0 ng was defined as methylated status, whereas the quantitative level equal to 0 was determined as unmethylated status. Afterwards, the qualitative data was used to measure the methylated status frequency in individual subgroups.

During screening, our results demonstrated OSCC showing *NID2* methylation with a higher frequency in the oral swab samples than oral rinse ones (20/22 [90.91%] vs. 34/43 [79.07%]; *P* > 0.05) as displayed in Table 1 and Fig. 3d. This value also presented the potential of *NID2 *methylation in screening OSCC in the oral rinse sample with 79.07% sensitivity and 100% specificity, whereas the screening OSCC capacity in the oral swab has 90.91% sensitivity and 100% specificity.

Among the total 43 matched OSCC cases between oral rinse and oral swab, only 22 patients were allowed to collect the samples with swabbing because of physical suffering. The results showed that 20 of 22 cases in oral swab were methylated (90.9%), whereas 17 of 22 cases of oral rinse were methylated (77.3%). Notably, both the cases defined that the unmethylation of oral swab samples were in the unmethylated cases of oral rinse samples, suggesting a higher sensitivity of oral swab compared with oral rinse. In addition, no statistically significant methylation level for the histological grade and clinical stage was noted in both samples collected by oral rinse or swab.

## Discussion

To date, OSCC is mostly detected at an advanced stage via the conventional gold standard methods, including clinical examination and biopsy [19]. Biopsy is a relatively painful and invasive procedure that affects patients both physically and psychologically. To reduce its adverse effects, an effective screening biomarker is needed. Because DNA is a stable macromolecule, DNA methylation is recognized as one of the candidate biomarkers for the early diagnosis of OSCC [20].

In this study, using our previous candidate gene analyzed from bioinformatics data, we developed an identification method for OSCC by utilizing a cancer-related methylated gene with noninvasiveness and high sensitivity. Moreover, the technique to score the methylation was improved by counting number that was easier to analyze. This technique can detect specific site methylation of *NID2* using both oral rinse and lesion swab samples from patients.

The present results of the bioinformatics calculation clearly show the correlation between increased methylation level and OSCC carcinogenesis. A comparison of the methylation level of cg22881914 of *NID2* in the oral tissue types lined up from normal to cancerous tissue and sequencing of bisulfite-conversed DNA from microdissection biopsy samples, demonstrated that hypermethylation occurred when the normal tissue became cancerous. Additionally, real-time PCR results indicated that the methylation was not detected in both epithelia of smokers and patients with OLP, which indicated that this marker was specific to cancerous epithelia. Furthermore, no significant difference was observed between the *NID2* methylation and clinical stage or histological grade of OSCC. Collectively, this suggests that at cg22881914 of *NID2*, the methylation had transition when normal epithelial cell completely transform to cancer cells.

We previously reported the *Alu* and *TRH* site-specific methylations for OSCC detection. However, the cutoff values of both markers have to be evaluated for cancer [14, 21]. In this study, the calculation of cg22881914 *NID2* methylation clearly indicated totally zero value in the normal oral epithelium, and eventually, the samples were used up to a concentration of 10 ng/μl. After we change to detect the quality level (met vs. unmet), this test was easier and more convenient to OSCC screening application.

Nidogen is a component found in the basement membrane. In particular, NID2 is one of nidogen family proteins that play a role in balance of integrity and stability of basement membranes through cooperation with laminin and collagen in the extracellular matrix [5, 13]. Moreover, its loss reportedly contributes to the development and progression of cancer, in which metastasis and invasion may be stimulated due to weakened cell–cell interaction [12, 20]. The relationship between* NID2* hypermethylation and cancer had been reported in terms of downregulation of NID2 expression in several cancers, suggesting tumor-suppressor activity of NID2 [9–11]. Recently, the reduction in the *NID2* mRNA and protein level has been determined in cancer tissue samples as well as in nude mice xenograft model. Further investigation using microarray analysis and other techniques revealed that *NID2* was hypermethylated, while its demethylation or overexpression could decrease many signs of cancer, such as proliferation, migration, invasion, including apoptosis [22]. Researches on the *NID2* methylation status and its dysfunction, which results in cancer, have been consistently published, increasing the reliability and importance of *NID2* [23–25]. Via both bioinformatic analysis and in vitro investigation, we confirmed the methylation of *NID2* related to cancer in cases of OSCC. Hence, it may have a tumor-suppressor function and be appropriately used as a biomarker for cancer detection.

*NID2* methylation had been suggested as being a potential biomarker in the diagnosis of OSCC using noninvasive samples such as saliva. The site of cg22881914 on *NID2* has been previously reported by Guerrero-Preston et al. with a very high sensitivity and specificity when a frozen tissue was tested [5]. However, when applied in the saliva, the specificity reduced to 21%, although the sensitivity was still the same at 87%. Although combination with HOXA9 promoter methylation for detection could improve the specificity up to 90%, the sensitivity was 50%, which was insufficient for screening. Nonetheless, using our methods, higher rates of sensitivity and specificity were noted; as such, the difference in the procedure used may have influenced the result. Furthermore, using a specific methylation probe, we could clearly distinguish the OSCC samples with the detectable methylated *NID2* level because no control sample had shown that level. This is a significant factor for effective screening and is different from the results of the study by Guerrero-Preston et al. using only quantitative methylation-specific PCR primers, which eventually needed cutoff evaluation [5].

We also improved the sensitivity rate using the swab approach in the sample collection, Matched oral rinse and oral swab OSCC samples show the higher frequency of methylated in oral swab than oral rinse group leading to an increase of up to 90%. The false-negative outcomes in two cases may have resulted from the improper clinical swab technique, in which the swab only covers necrotic tissue over the lesion. Moreover, this swab approach could be used only in patients who presented lesions but not in those without lesions.

Although *NID2* methylation is a promising marker for cancer screening, a larger sample size is necessary, and a different cohort should be used for unequivocal results. Because NID2 is a cell-adhesion protein on the basement membrane, *NID2* methylation could lead to a loss of NID2 expression, resulting in a false negative detection in epithelial dysplasia or non-invasive cancerous lesion. On the other hand, a false positive result is likely potential in examination applied to the sample of some basement membrane damageable mucosal lesions. In general, epigenetic change is always related to some systemic conditions/diseases [26, 27]. To minimize potential bias, relative samples that might be involved should also be included in the investigation, such as abnormal oral lesions or other abnormal systemic conditions/diseases. For the technical aspect, the level of *NID2* methylation is measured using real-time PCR based technique. It is validated using a standard universal methylation DNA of the positive control. This validation among laboratories can minimize the technical limitation that might influence an error on methylation level.

## Conclusion

In this study, the methylation at cg22881914 of *NID2* was an altered specific condition that was observed in OSCC but not in normal mucosa, smokers and OLP epithelium. Therefore, the assessment of methylation status in *NID2* was a potential OSCC screening method that could be used to detect oral cancer in sample collected from invasive method such as oral rinse and swab approaches.

## Data Availability

All data generated or analyzed and its supplementary information files during this study are included in this published article.

## Abbreviations

AUC: Area under curve
cg: CG dinucleotide
FFPE: Formalin-fixed paraffin embedded tissue
GEO: Gene expression omnibus
H&E: Hematoxylin and eosin (H&E)
HNSCC: Head and neck squamous cell carcinoma
NCBI: National center for biotechnology information
NID2: Nidogen-2
OSCC: Oral squamous cell carcinoma
PBS: Phosphate-buffered saline
PCR: Polymerase chain reaction
ROC: Receiver operating characteristic
SCC: Squamous cell carcinoma
